# Change of histone H3 lysine 14 acetylation stoichiometry in human monocyte derived macrophages as determined by MS-based absolute targeted quantitative proteomic approach: HIV infection and methamphetamine exposure

**DOI:** 10.1186/s12014-023-09438-5

**Published:** 2023-10-25

**Authors:** Katarzyna Macur, Andrew Schissel, Fang Yu, Shulei Lei, Brenda Morsey, Howard S. Fox, Pawel Ciborowski

**Affiliations:** 1 Core Facility Laboratories, Intercollegiate Faculty of Biotechnology UG & MUG, University of Gdańsk, Gdańsk, Poland; 2 Department of Pharmacology and Experimental Neuroscience, University of Nebraska Medical Center, Omaha, NE USA; 3 Department of Neurological Sciences, University of Nebraska Medical Center, Omaha, NE USA; 4 Department of Biostatistics, University of Nebraska Medical Center, Omaha, NE USA

**Keywords:** HIV, Methamphetamine, Histone H3 lysine 14 acetylation (H3K14Ac), Proteomics, Mass spectrometry, Absolute quantification, Liquid chromatography

## Abstract

**Background:**

Histones posttranslational modification represent an epigenetic mechanism that regulate gene expression and other cellular processes. Quantitative mass spectrometry used for the absolute quantification of such modifications provides further insight into cellular responses to extracellular insults such as infections or toxins. Methamphetamine (Meth), a drug of abuse, is affecting the overall function of the immune system. In this report, we developed, validated and applied a targeted, MS-based quantification assay to measure changes in histone H3 lysine 14 acetylation (H3K14Ac) during exposure of human primary macrophages to HIV-1 infection and/or Meth.

**Methods:**

The quantification assay was developed and validated to determine H3K14Ac stoichiometry in histones that were isolated from the nuclei of control (CIC) and exposed to Meth before (CIM) or/and after (MIM) HIV-infection human monocyte-derived macrophages (hMDM) of six donors. It was based on LC–MS/MS measurement using multiple reaction monitoring (MRM) acquisition of the unmodified and acetylated form of lysine K14 of histone H3 ^9^KSTGGKAPR^17^ peptides and the corresponding stable isotope labeled (SIL) heavy peptide standards of the same sequences. The histone samples were propionylated (Poy) pre- and post- trypsin digestion so that the sequences of the monitored peptides were: K[Poy]STGGK[1Ac]APR, K[Poy]STGGK[1Ac]APR-heavy, K[Poy]STGGK[Poy]APR and K[Poy]STGGK[Poy]APR-heavy. The absolute amounts of the acetylated and unmodified peptides were determined by comparing to the abundances of their SIL standards, that were added to the samples in the known concentrations, and, then used for calculation of H3K14Ac stoichiometry in CIC, CIM and MIM hMDM.

**Results:**

The assay was characterized by LLOD of 0.106 fmol/µL and 0.204 fmol/µL for unmodified and acetylated H3 ^9^KSTGGKAPR^17^ peptides, respectively. The LLOQ was 0.5 fmol/µL and the linear range of the assay was from 0.5 to 2500 fmol/µL. The absolute abundances of the quantified peptides varied between the donors and conditions, and so did the H3K14Ac stoichiometry. This was rather attributed to the samples nature itself, as the variability of their triplicate measurements was low.

**Conclusions:**

The developed LC–MS/MS assay enabled absolute quantification of H3K14Ac in exposed to Meth HIV-infected hMDM. It can be further applied determination of this PTM stoichiometry in other studies on human primary macrophages.

**Graphical Abstract:**

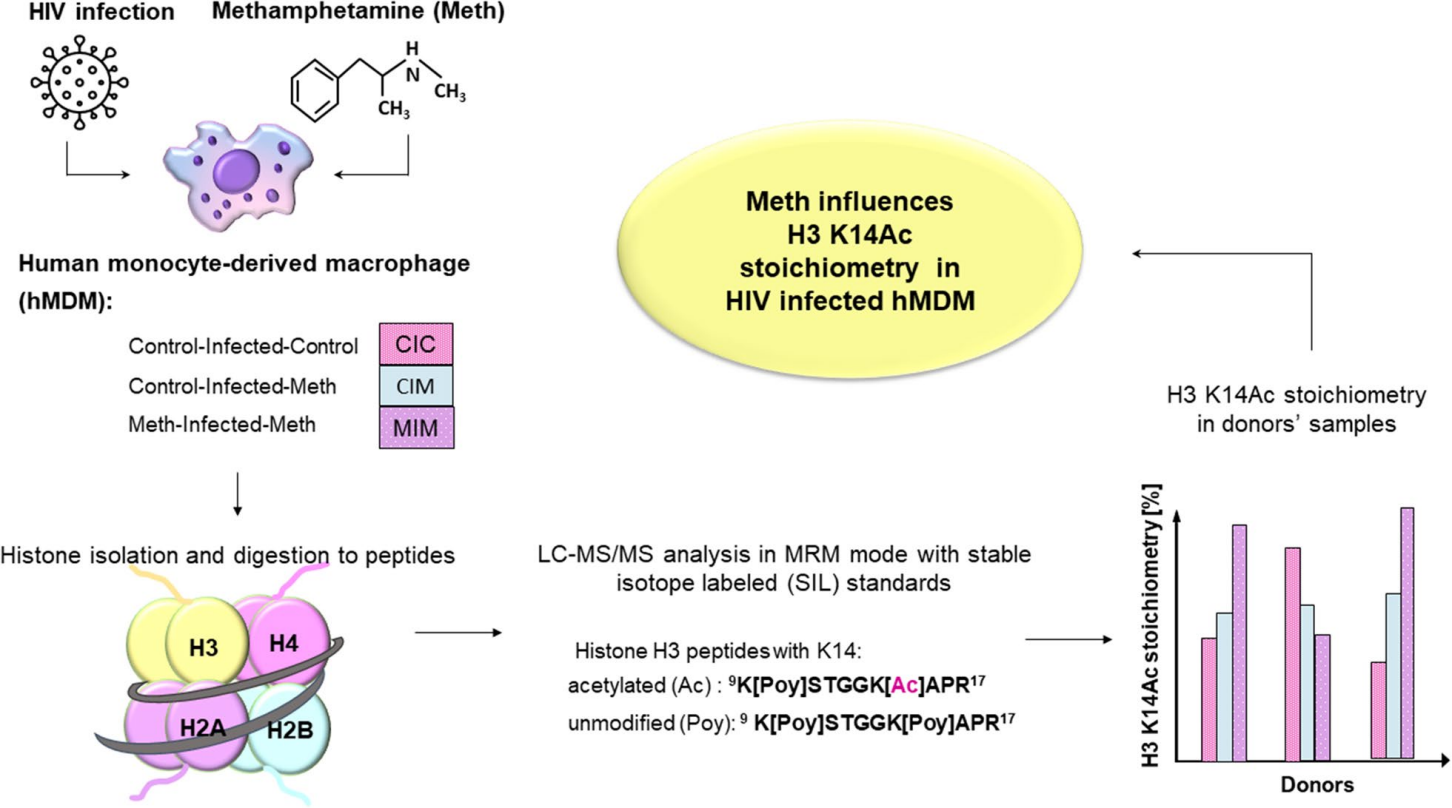

**Supplementary Information:**

The online version contains supplementary material available at 10.1186/s12014-023-09438-5.

## Background

Post-translational modifications (PTMs) of histones are one part of highly complex epigenetic regulatory mechanisms [1] regulating functions of the proteins in spatial and temporal manners [2]. Cells use this regulatory machinery for rapid and dynamic response to different intracellular and environmental stimuli suggesting interdependency of these modifications also working in a “code” type combinatorial manner. As indicated in the literature, hPTMs regulate gene expression, and the type and location of different PTMs can activate or silence this process. Moreover this regulation is a dynamic process and hPTMs can be short lived in their function before returning to the steady state [3–6]. Therefore, stoichiometry of histone PTMs (hPTMs) at any time point of experimental manipulation might be of crucial importance. Our understanding is that modifications in PTMs’ changing from baselines is not necessarily all or nothing.

Changes in hPTMs are orchestrated by histone modifying enzymes and reflect cells’ response to a variety of stimuli. These stimuli can be related to differentiation, response to environmental changes including insults by all types of infections [7], toxins, as well as establishing latent viral infection, i.e., HIV-1 [8]. In our study we investigated impact of HIV infection and Meth exposure on human monocyte-derived macrophages (hMDMs). Devastating effects of Meth and HIV-1 infection are biologically complex processes and are strongly associated. Meth predisposes users for risky sexual behaviors, which may lead HIV-1 infections. Moreover, it was observed that the Meth users who are also HIV-1 positive, have higher viral loads than non-users. Additionally, immune-modulatory properties of Meth that manifests by eg. regulation of cytokines and chemokines, contributes to increase in viral transmission, replication and immune dysfunction. These factors lead to disease progression of HIV-1 infected Meth users. Macrophages, investigated in our study, play important role in HIV-1 infection and Meth was shown to enhance HIV infection [9]. However, molecular mechanisms underlying these effects are vastly unknown, thus we “primed” macrophages with Meth before infection and treated infected cells with Meth to investigate the differences. Our observations indicate that differentiation of monocytes to MDMs is also accompanied with changes in hPTMs; however, these changes require further studies using quantitative proteomics. We have also found that exposure of mononuclear phagocytes (MP) to methamphetamine (Meth) leads to changes in several hPTMs in MP (unpublished). How these changes correlate with changes in phenotype or function is not yet known. The effect of Meth on phenotypic profile of MP has been documented by number of reports [10–13]. The molecular mechanisms underlying these effects are not known; thus, at this time, it requires broader systems biology approaches before specific hypotheses are put forward.

Mass spectrometry proved to be an efficient tool for analysis of PTMs of proteins [14]. Methods for global PTMs mapping, e.g., label-free quantification, stable isotope labelled amino acids in cell culture (SILAC) or tandem mass tags (TMT), enable detection of multiple PTMs in one experiment, but some low abundant PTMs may not be detectable. The experimental evidence of acetylation abundance shows that this modification often occurs in low abundance. Therefore, targeted methods with stable isotope labeled (SIL) standards, especially with multiple reaction monitoring (MRM) mass spectrometry (MS) data acquisition, sometimes referred to as selected reaction monitoring (SRM), seem to be the most suitable methodology for reliable quantification of acetylation stoichiometry. The MRM mode of acquisition is characterized by high selectivity, sensitivity, accuracy, and broad dynamic range, that detects lower abundance changes. In this study, we used the AQUA approach [15, 16] to determine absolute quantities of post-translationally modified histones by acetylation and unmodified lysine K14 of histone H3. Based on that result we calculated the stoichiometry of this PTM. In AQUA methodology, the absolute levels of protein are defined based on the absolute levels of its proteotypic peptides. For accurate measurements of the peptides, the samples are spiked with a known amount SIL standard peptides of the same sequence and analyzed by liquid chromatography-tandem mass spectrometry (LC–MS/MS) in the MRM mode. Comparison of the resulting peak areas of the native and the spiked-in reference peptide of known concentration enables us to determine absolute quantity of the native peptide in the sample. Because native and SIL peptides are chemically identical in terms of chromatographic behavior, ionization efficiency, and fragmentation mechanism in MS, they are directly comparable; and their abundances are proportional to their levels in the sample [16]. In the case of determining PTM stoichiometry using AQUA approach, it is necessary to synthetize two types of SIL standard peptides: one corresponding to a PTM modified peptide and one for the unmodified peptide of the investigated protein. Known quantities of these two kinds of SIL peptides are then added to the proteolytically digested sample of interest. Relative intensities of both, PTM-modified and unmodified, native, and SIL peptides are further measured in the LC–MS/MS analysis in the MRM mode and absolute abundance of the endogenous peptides is calculated. Finally, the stoichiometry of the given PTM is determined as a percentage of the modified peptide abundance to sum of both, modified and unmodified peptides abundances [17].

**Fig. 1:**
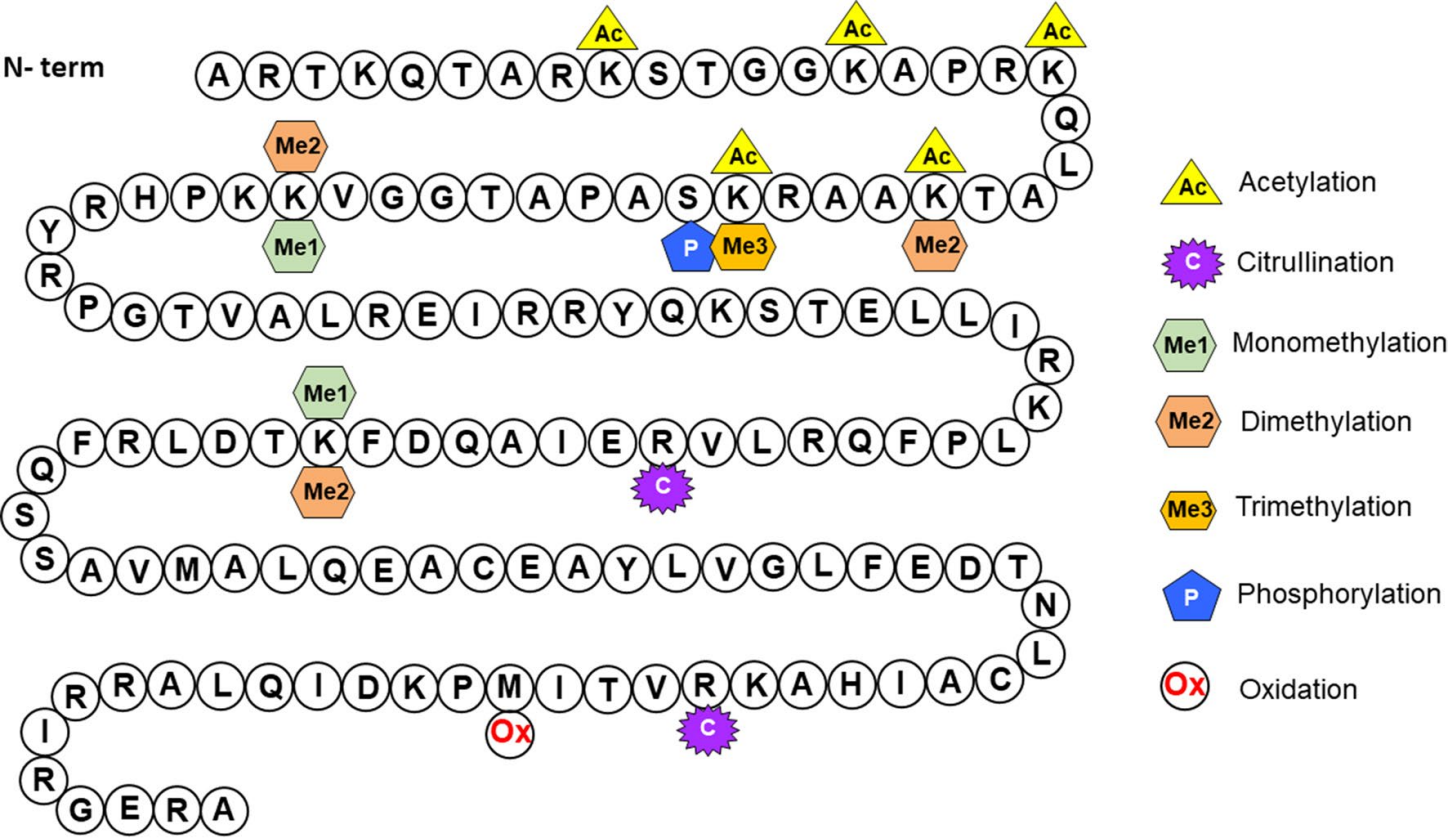
PTMs of histone H3 of resting hMDM found by Olszowy et al. [18]

A previous study from our group resulted in a qualitative map of hPTMs of the resting hMDM [18] (Fig. 1). In this study, we chose one of them—histone H3 acetylation (Ac) of lysine 14 (K14)—for targeted quantification in the HIV-infected and Meth exposed hMDMs, based on its biological relevance. H3 K14Ac was shown to be involved in important biological processes, e.g. in regulating DNA damage checkpoint [19]. H3 K14Ac was also found along with K9Ac to be involved in regulation of locomotor behavior induced by cocaine, another drug of abuse, and resilience to social stress [20]. Using histone K14Ac, we show that absolute quantification based on mass spectrometry provides reliable measurements of changes in hPTMs. Application of our methodology will have an impact on understanding the function of mammalian homologs.

## Materials and methods

### Patients’ samples

The hMDMs used in this study were obtained by differentiating the monocytes from six healthy, HIV-1-, HIV-2- and hepatitis- seronegative human donors. The leukapheresis from normal donors was done in core facilities, similarly as it was in Macur et al.[12] and Grabowska et al. [21]**.**

### Cell culture and HIV infection

Monocytes were obtained from leukapheresis and purified by counter-current centrifugal elutriation as previously described [22]. Differentiation, infection with HIV-1, and exposure to Meth are described in [12]. Samples of collected cells were CIC (control-infected-control), CIM (control-infected-Meth exposed) and MIM (Meth exposed-infected-Meth exposed). Cells from these three conditions were used to isolate histones.

### Histone isolation

Digested histone CIC, CIM, and MIM samples were derived from the six human donors included in this study and designated as D160, D164, D222, D406, D410, D423. A separate set of histones from cultured hMDMs from another 10 different donors was pooled together to create biological sample matrix for the development and validation of the LC–MS/MS method.

The cell pellets were washed in nuclear isolation buffer (NIB) without NP-40 Alternative (Millipore, Bedford, MA, USA) in the 1:10 cell pellet to buffer ratio (v/v). Supernatant was removed by centrifugation at 700 rcf for 5 min. The cell pellets were placed on ice and lysed by adding NIB with 0.2% NP-40 Alternative in the 1:10 cell pellet to buffer ratio (v/v). The cell lysates were left on ice for 5–10 min and then centrifuged at 1000 rcf for 5–10 min at 4 ℃. The nuclei pellets were then washed by gently resuspending them in 500 µL NIB complete without NP-40 Alternative at 1:5 (v/v) ratio. Next, the samples were centrifuged at 1000 rcf for 5 min at 4 ℃ then supernatants were removed. The wash step was repeated two times to completely remove NP-40 Alternative. The cell nuclei were resuspended in 1:5 (v/v) chilled 0.2 M H_2_SO_4_ by gentle pipetting. The samples were incubated with constant rotation for 2–4 h at 4 ℃. Then, they were centrifuged at 3400 rcf at 4 ℃ for 5 min. The supernatants were transferred to new tubes. The centrifugation and transfer steps were repeated twice to remove any insoluble material. Next, the histones were precipitated by adding chilled 100% trichloroacetic acid (TCA) (Sigma-Aldrich, St. Louis, MO, USA) with ½ volume of TCA to the collected supernatant to obtain a final TCA concentration of 33%; the samples were mixed by pipetting. The mixtures were incubated on ice for at least 1 h. Then, the mixtures were centrifuged at 3400 rcf for 5 min; and the supernatants were carefully (without scraping the sides or the pellet) removed by aspiration. By using a glass Pasteur pipette, the tubes were rinsed with ice-cold acetone + 0.1% HCl (both Sigma-Aldrich, St. Louis, MO, USA), centrifuge at 3400 rcf for 2 min; and supernatants were carefully aspirated. The rinse and centrifuge step were repeated using 100% ice-cold acetone. The obtained histone pellets were dried with a vacuum centrifuge. Then, they were dissolved with 100 µL ddH_2_O (double distilled water), centrifuged at 3400 rcf for 2 min, and the supernatants were transferred to new tubes. The samples were dried in a vacuum centrifuge. The histone pellets were stored in – 80 ℃ until further use.

## Histone samples preparation for bottom-up targeted absolute MS proteomic quantification

### Histone propionylation pre-digestion

The samples containing isolated histones were dissolved in 40 µL of 50 mM NH_4_HCO_3_ (Sigma-Aldrich), pH 8.0. Then, a propionylation reagent was freshly prepared by mixing propionic anhydride (Sigma-Aldrich) with acetonitrile (Honeywell Burdick & Jackson, Morristown, NJ, USA) in the ratio 1:3 (v/v). Caution should be taken when working with propionic anhydride as it is a combustible and corrosive substance. The propionylation reagent was added to samples in 1:4 (v/v) ratio, after which NH_4_OH (Sigma-Aldrich) was quickly added to the samples with a ratio of 1:5 (v/v) to re-establish pH 8.0 to the solution. The samples were mixed immediately by vortexing, the pH was checked, and they were incubated at room temperature for 15 min. Next, the samples were dried, resuspended in 40 µL of 50 mM NH_4_HCO_3,_ and the propionylation step was repeated to ensure complete derivatization, as it was done by Sidoli et al. [23].

### Proteolytic digestion with trypsin

The histone samples were resuspended in 50 mM NH_4_HCO_3_ to achieve an optimal concentration of 1 μg/μL or trypsin (Trypsin Gold MS Grade, Promega, Madison, WI, USA) was added to the samples at a weight/weight (wt/wt) 1:10 ratio. They were incubated at 37 ℃ for 6–8 h. Digestion was stopped by freezing in − 80 ℃, and the samples were dried in a SpeedVac.

### Histone propionylation post-digestion

The histone tryptic digests were resuspended in 30 µL of 100 mM NH_4_HCO_3_. Then, histone propionylation was performed as described in the *Histone propionylation pre-digestion* section, and the propionylated samples were dried.

### Sample clean-up using mixed cation exchange (MCX)

The dried samples were acidified by resuspending it in 0.4% formic acid (LC/MS Optima, Thermo Fisher Scientific) in water (LC–MS grade, Honeywell Burdick & Jackson). The Oasis MCX cartridges (Waters, Milford, MA, USA) were equilibrated with 1 mL of 1:1 methanol: water (both LC–MS grade, Honeywell Burdick & Jackson). Then the samples were applied to the MCX cartridges. After that the cartridges were washed with 1 mL of 5% methanol/0.1% formic acid in water and then with 1 mL of 100% methanol. The bound peptides were eluted with 1 mL of 50 µL of 28% NH_4_OH solution (≥ 99,99% trace metal basis, Sigma-Aldrich) in 950 µL of methanol. The eluted peptides were dried in SpeedVac.

### MS sample preparation

The samples were resuspended in 0.1% formic acid in water, and the peptide concentrations were determined using NanoDrop analysis at 230 nm.

For development and validation of the absolute quantification (AQUA) LC–MS/MS method, the prepared histone CIC, CIM and MIM donors’ samples, as well as the pooled histone matrix sample, were resuspended to 0.5 µg/µL with 0.1% formic acid in water. An aliquot of each of the investigated donor samples from all analyzed conditions (CIC, CIM, MIM) were pooled together to create a quality control (QC) sample that was run in each batch with the particular donor samples. The AQUA approach involves the use of SIL standards of the identical sequence to the investigated peptides to determine the amounts of the endogenous analytes. Following the recommendations in the literature to determine the histone H3 K14 acetylation stoichiometry using AQUA approach [15, 16] there were two types of internal standard peptides synthetized—an acetylated (Ac) and nonacetylated K14 that also included the ^13^C/^15^N isotopically labelled C-terminal arginine (R) residue (heavy). This resulted in 10 Da shift in molecular weight (MW) compared to the native peptide present in the investigated samples. There were also Ac and nonacetylated unlabeled peptides synthetized (light) that matched the sequence with those endogenous native peptides occurring in the donors’ samples, which were used during the method validation as internal standards for creating a reverse calibration curve. Both, heavy and light peptides (AQUA QuantPro standards, 5 pmol/µL in 50/50 ACN/water, custom synthetized by Thermo Fisher Scientific), were of > 97% purity and > 99% isotopic enrichment as recommended by Hoofnagle et al. [24]. Moreover, the nonacetylated K residues of both heavy and light peptides, i.e. K9 in acetylated or K9 and K14 in nonacetylated peptides, were propionylated (Poy). Because the target samples were propionylated during sample preparation, the propionyl groups at the K residues that were not post-translationally modified were introduced. Therefore, there were four histone H3 peptide standards used in the present study: K9[Poy]K14[Ac], K9[Poy]K14[Ac]-heavy, K9[Poy]K14[Poy], and K9[Poy]K14[Poy]-heavy. The sequences of each of the peptides as well as their calculated molecular weight is presented in Table 1. Prior to the LC–MS/MS analyses and to achieve final concentration of 50 fmol/µL SIL standards in the sample as recommended in Clinical Proteomic Tumor Analysis Consortium (CPTAC) guidelines, the donors’ CIC, CIM, and MIM samples were spiked with SIL standards of the histone H3 K9[Poy]K14[Poy]-heavy and K9[Poy]K14[Ac]-heavy in the volumes that comprised 10% of the sample volume [25]. The histone matrix samples used during the LC–MS/MS method development and validation process were also spiked with K9[Poy]K14[Poy]-heavy and K9[Poy]K14[Ac]-heavy peptides in the volumes that comprised 10% of the sample volume, to obtain seven dilutions of the final concentrations of 0.5, 1.25, 5, 50, 500, 1250, and 2500 fmol/µL and a QC of 12.5 fmol/µL of heavy peptides. The matrix blank samples (0 fmol/µL of the heavy peptides concentration) were spiked with the same volume of the 50/50 ACN/water instead of the heavy peptides’ dilution. All the histone samples for method development and validation were additionally spiked with 100 fmol of non-labelled H3 K9[Poy]K14[Poy] and K9[Poy]K14[Ac] peptides that served as internal standards during creation of the reversed calibration curves, similarly as in [26].

**Table 1:** Histone H3 peptides analyzed in the present study (the amino acid, on which the SIL was introduced, is depicted bold and underlined)

Peptide name	Calculated monoisotopic molecular mass	Amino acid sequence	Precursor ion *m/z*	Precursor ion charge state	Product ion *m/z*	Product ion type	Product ion charge state	MRM transition *m/z*	MRM transition name
K9[Poy]K14[Ac]	999.5587	K[Poy]STGGK[1Ac]APR	500.3	+ 2	728.4	y7	+ 1	500.3/728.4	MRM1
			500.3	+ 2	815.4	y8	+ 1	500.3/815.4	MRM2
			500.3	+ 2	627.6	y6	+ 1	500.3/627.6	MRM3
K9[Poy]K14[Ac]-heavy	1009.5669	K[Poy]STGGK[1Ac]AP**R**	505.3	+ 2	738.4	y7	+ 1	505.3/738.4	MRM4
			505.3	+ 2	825.4	y8	+ 1	505.3/825.4	MRM5
			505.3	+ 2	637.6	y6	+ 1	505.3/637.6	MRM6
K9[Poy]K14[Poy]	1013.5744	K[Poy]STGGK[Poy]APR	507.4	+ 2	742.3	y7	+ 1	507.4/742.3	MRM7
			507.4	+ 2	829.3	y8	+ 1	507.4/829.3	MRM8
			507.4	+ 2	641.5	y6	+ 1	507.4/641.5	MRM9
K9[Poy]K14[Poy]-heavy	1023.5826	K[Poy]STGGK[Poy]AP**R**	512.3	+ 2	752.4	y7	+ 1	512.3/752.4	MRM10
			512.3	+ 2	839.4	y8	+ 1	512.3/839.4	MRM11
			512.3	+ 2	651.4	y6	+ 1	512.3/651.4	MRM12

### LC–MS/MS analyses in the MRM mode

Histone tryptic digests were analyzed using reverse phase ultra-high-performance LC–MS/MS technique on an Acquity I-Class UHPLC (Waters) coupled with a QTRAP 6500+ mass spectrometer (SCIEX, Framingham, AM, USA), and controlled by Analyst software (SCIEX). The samples (4 μL) were injected in three technical replicates via an autosampler kept at 8 ℃ into the Omega (1.6 μm, PS C18, 100 × 2.1 mm, 100 Å) reversed-phase high performance liquid chromatography (RP-HPLC) column with SecurityGuard ULTRA Cartridge (Phenomenex, Torrance, CA, USA). 0.1% (v/v) formic acid in water (solvent A) and 0.1% (v/v) formic acid in acetonitrile (ACN) (both LC–MS grade from Honeywell Inc.) at a flow rate of 0.2 mL/min were used for separation of the samples’ components using a linear gradient of 5% to 50% solvent B in 25 min, then ramped to 95% in 3 min, after which the column was re-equilibrated with 5% solvent B for 10 min. Electrospray ionization of the samples was achieved in TurboV Ion Source (SCIEX) operated at a positive 5,5 kV spray voltage, with curtain gas at 35 psi, ion source gas 1 and 2 at 45 psi, temperature 450 ℃, CAD collision gas (CAD) flow medium and then analyzed in MRM mode. We used an absolute quantification (AQUA) approach [15] to determine the histone H3 K14Ac absolute abundance and stoichiometry in our samples. The AQUA strategy employs liquid chromatography (LC) separation with MRM detection, and the levels of endogenous peptide are determined by comparison to the signal of stable isotope labelled internal standard of this peptide that was added in a known amount to the sample [15, 16]. To achieve this goal, we developed the MRM assays for four peptides: K9[Poy]K14[Ac], K9[Poy]K14[Ac]-heavy, K9[Poy]K14[Poy] and K9[Poy]K14[Poy]-heavy. The MRM assays for quantification of the studied histone H3 peptides and their SIL standards were optimized manually by directly infusing to the QTRAP 6500+ mass spectrometer the AQUA QuantPro (> 97% purity and > 99% isotope enrichment from Thermo Fisher Scientific) standard solutions of the synthetic peptides of the above-mentioned sequences. The *m/z* of +2-charged precursor ions were selected from the enhanced MS (EMS) scan, while three of the +1 charged product ions *m/z* were selected based on their intensity observed during the enhanced product ion (EPI) MS scan performed for each of the analyzed peptides. After checking for specificity of the MRM transitions for K9[Poy]K14[Poy]APR and K9[Poy]K14[1Ac]APR peptides, we chose three of the best performing peptides for the final LC–MS/MS method, as indicated in CPTC Assay Development Guidelines ver.1.0 [27]. The details of the peptides analyzed in this study and their MRM transitions are presented in Table 1. The MRM dataset from the presented study was submitted to PASSEL repository (PeptideAtlas SRM Experiment Library, accession number PASS03789) [28] and is available under the accession number PASS03789 (https://db.systemsbiology.net/sbeams/cgi/PeptideAtlas/PASS_View?identifier=PASS03789) The MS parameters were individually optimized for each MRM transition, e.g., de-clustering potential (DP), collision energy (CE), entrance potential (EP), and cell exit potential (CXP); these are listed in Additional file 1: Table S1. Manually optimized MRM transitions list was imported to Skyline-daily ver. 21.1.1.327 along with the MS files from LC–MS/MS runs of the samples to perform peak extraction and data processing [29, 30]. Human histone H3 (UniProt accession number P68431) sequence in canonical FASTA format was downloaded from UniProt [31] along with the manually optimized MRM transitions to create targets list in Skyline. The following Skyline Peptide Settings were applied: digestion with trypsin [KR|P] with a maximum of 5 missed cleavages; peptides with 8 to 10 amino acids (AA) and 5 N-terminal AA excluded; structural modifications: cysteine carbamidomethylation, propionylation of lysine and N-term, lysine acetylation with up to 4 variable modifications, 1 maximum loses, heavy isotope label ^13^C_6_^15^N_4_ at C-terminal arginine and light internal standard (for reverse calibration curve creation) or heavy (for separate donor’s samples processing). Skyline Transition Settings included: monoisotopic precursor and product masses; SCIEX collision energy; for filtering the peptides: +2 charged precursor ions, +1 product ions, from ion 1 to last ion were chosen with N-terminal proline as special ions. A range of 300 m/z to 1500 m/z with a method match tolerance of 0.25 m/z was chosen for the instrument. Peptides’ peak areas were calculated in Skyline software as a sum of peak areas of MRM transitions of their corresponding ions. Light to heavy peak area ratios for each of the modified and unmodified peptide and the known concentration of the added heavy peptide standards (K9[Poy]K14[Ac]-heavy and K9[Poy]K14[Poy]-heavy) were used by the software for calculation of the absolute quantities (fmol/µL) of the endogenous K9[Poy]K14[Ac] and K9[Poy]K14[Poy] peptides in the donors’ CIC, CIM, and MIM samples (Additional file 1: Table S4).

### H3 K14Ac stoichiometry calculation

The absolute abundances of native modified (K9[Poy]K14[Ac]) and unmodified (K9[Poy]K14[Poy]) peptides determined in Skyline (Additional file 1: Table S4) were then applied for calculation of the H3 K14Ac stoichiometry in CIC, CIM, and MIM donors’ samples (Additional file 1: Table S6) using the following equation proposed by Prus et al. [17]:

$$Stoichiometry \left(\%\right)=\frac{{Abs}^{native\_mod}}{{{Abs}^{native\_mod}+Abs}^{native\_unmod}} \times 100$$

where, Abs^native_mod^ is absolute abundance of native modified peptide, Abs^native_unmod^ is absolute abundance of native unmodified peptide. The calculated H3K14Ac stoichiometries for three technical replicates of each of analyzed conditions (CIC, CIM, MIM) for each donor were averaged to obtain average H3K14Ac stoichiometries in CIC, CIM, and MIM samples for donors. Additionally, the CIC, CIM, and MIM H3K14Ac stoichiometries for all six donors were also averaged, to obtain average stoichiometries for each of the analyzed conditions in all donors’ samples together. The SDs and % coefficient of variation (CVs) were calculated as well (Additional file 1: Table S6). These average H3K14Ac stoichiometries, for both, all donors together and each one separately, for CIC, CIM, and MIM conditions, are presented in a bar chart created using features available in Excel software (Microsoft Inc.) (Fig. 3).

The H3 K14Ac stoichiometry between CIC and CIM, CIC, and MIM, and CIM and MIM conditions were compared using a two-sample unequal variance T-test. If the p value was < 0.05, the differences were considered statistically significant.

### Validation of the LC–MS/MS method in the MRM mode

Design of the validation procedure of the quantitative proteomic LC–MS/MS method in the MRM mode was based on the Assay Development Guidelines CPTAC Assay Development Working Group Version 1.0 [27] of the CPTAC of National Institutes of Health, National Cancer Institute. We used Skyline software for data processing and analysis as proposed there. Additionally, we created for our assay for research use; so to make it fit-for-purpose, we also applied recommendations of Carr et al. [32]. According to [27], we prepared the dilutions of the standard peptides in the matrix of interest for creating the multipoint calibration curve of seven different concentrations spanning from 0.5 fmol/uL to 2500 fmol/uL, QC at 12.5 fmol/uL and a matrix blank. The matrix blank was injected in nine replicates; the other concentrations were injected in five replicates each. The matrix for calibration curve was part of the experiment and the matrix of the target samples was the same: it constituted of the digested human histone extract propionylated pre- and post-digestion (from different donors, other than the target samples for analysis). We used the same protocol for sample preparation for both, the matrix for calibration curve creation and the target CIC, CIM, and MIM samples, with the exception that the MDM for the matrix samples were not HIV-infected and Meth exposed. The analytes quantified in this study—human histone H3 peptides K9[Poy]K14[Ac] and K9[Poy]K14[Poy] were likely to be occurring in the matrix in the detectable range. For that reason, we used a reverse calibration curve approach, where the matrix is spiked with SIL standards at variable concentrations and the native analyte—as an internal standard—at the same concentration.

Features available in Skyline software were used to prepare the reversed calibration curves for H3 K9[Poy]K14[Poy]-heavy and K9[Poy]K14[Ac]-heavy peptides and calculate figures of merit: lower limit of detection (LLOD) and lower limit of quantification (LLOQ) to determine sensitivity of the assay. The reversed calibration curves were created by plotting nominal analyte concentration (fmol/µL) versus heavy:light peak area ratio for each of the heavy peptides studied (Additional file 2: Figure S1 for K9[Poy]K14[Ac]-heavy and 2S for K9[Poy]K14[Poy]-heavy). The Skyline Peptide Settings and Transitions Settings applied for were the same as described in the “*LC–MS/MS analyses in the MRM mode*” paragraph with the exceptions listed below. We applied linear in Log space regression fit (none weighing) to create those reversed calibration curves. The max LLOQ bias (relative error) and LLOQ CV were set to 15% and qualitative ion ratio threshold set to 30%. The LOD was calculated from the blanks injected before the calibration curve as the average plus 3 times the standard deviation (SD) of the signal of the blank (3 × SD of the blank). The LLOQ was determined as “the lowest concentration of peptide at which the imprecision of the assay (expressed as the CV) is < 20% “ [27]. The linearity of the calibration curves was determined using linear regression and calculation of the Pearson’s determination coefficient R^2^ [26]. The accuracy and precision were also calculated, as a ratio of back-calculated concentration to the actual analyte concentration, and %CV of the replicate measurements of the same standard concentration, respectively. The calibration points had to be within 85–115% of the theoretical concentration or 80–120% at the LLOQ. The upper limit of quantification (ULOQ) was determined as the highest concentration measured whereas, a linear response between LLOQ and ULOQ was defined as a linear range [27]. The selectivity of the method was evaluated by visual examining the potential presence of the interferences in the matrix blank samples in the retention time region of the analytes (heavy peptides). To ensure that there is no interference on the analytes detection by other substances present in the samples, the specificity of the assay was assessed. At first, already on the MRM transitions development stage of the study, we used the MS Product feature of ProteinProspector software [33] to inspect whether the MRM transitions are unique for the peptides of interest and do not overlap with MRM transitions of the isobaric peptide K9[Ac]K14[Poy] that might be also present in the investigated samples. Then, the MRM transitions’ ratios for each of the analyzed peptides were determined to evaluate the specificity of the assay. The peak areas for each transition were normalized to total peak area of all transitions for analyte (Percentage peak areas) and compared between each other to obtain MRM transitions ratios. The mean, SD and %CV for each MRM transition ratio were also calculated for both the standard dilutions above the LLOQ and donors’ samples. The MRM transition ratios for all samples of concentrations above LLOQ should be within 30% from the mean.

The document grid option in Skyline was used to present the results of the LC–MS/MS method validation on peptide and MRM transitions levels, which are presented in the Additional file 1: Table S2 and S3.

Heavy to light peak area ratios for each of the modified and unmodified peptide and the concentration of the added light peptide standards (K9[Poy]K14[Ac] and K9[Poy]K14[Poy]) were used by the software for calculation of the absolute quantities (fmol/µL) of the K9[Poy]K14[Ac]-heavy and K9[Poy]K14[Poy]-heavy peptides in the series of the SIL standards dilutions used for reversed calibration curve preparation.

## Results

### *Absolute quantification (AQUA) of histone H3 lysine K14 acetylation (Ac) using targeted MRM-based* proteomic analysis and SIL standards

**Fig. 2:**
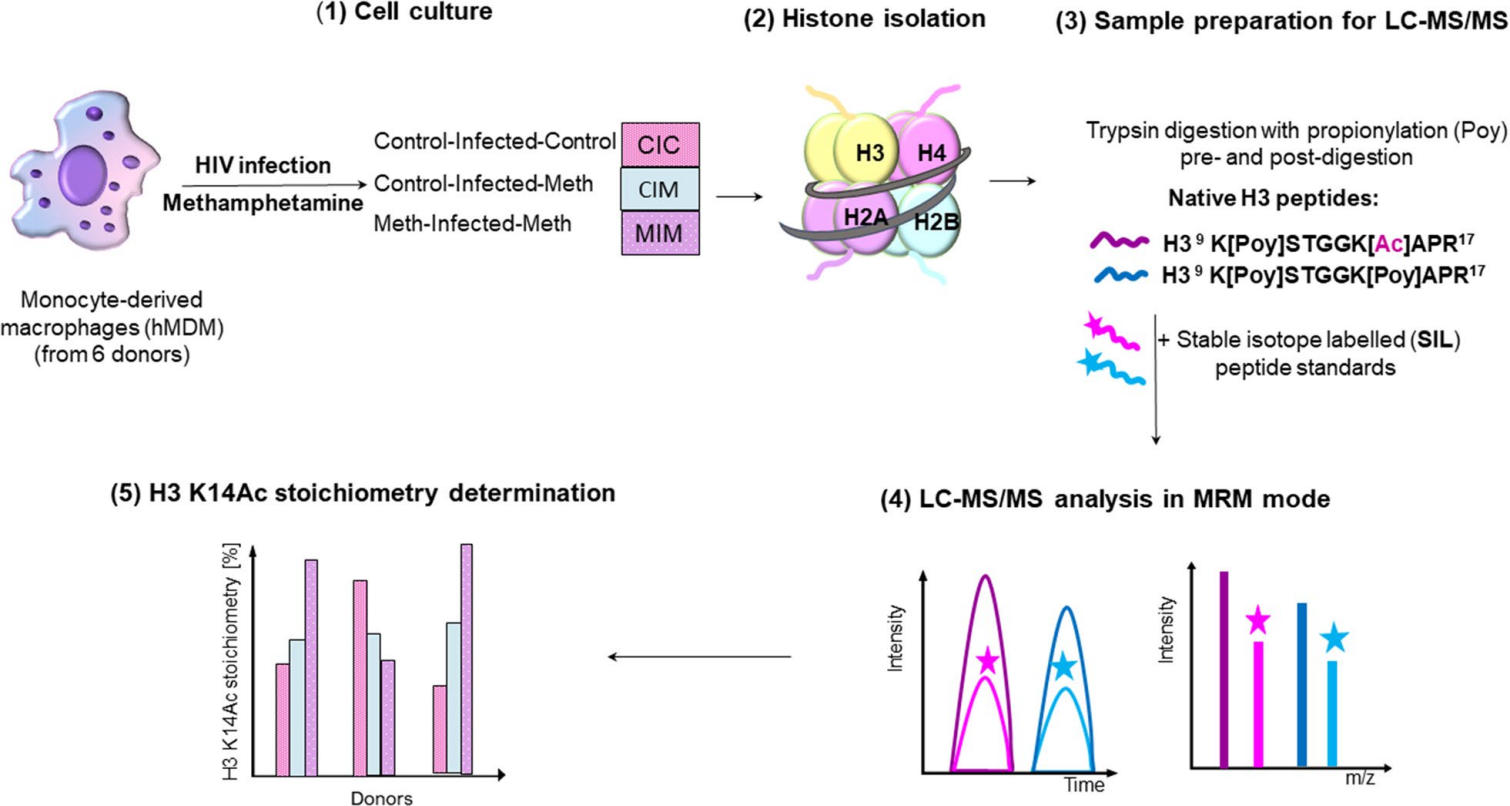
Experimental workflow applied to determine histone H3 K14 acetylation stoichiometry in CIC, CIM and MIM hMDM obtained from six donors

Global and untargeted identification of hMDM hPTMs have been published previously [18]. The summary of PTMs found in this study on histone H3 in primary macrophages is summarized in Fig. 1. At that time, we did not quantify the extent of histone H3 K14 acetylation. Based on the results of the untargeted profiling of the histone H3 PTMs, in this report we chose the acetylation (Ac) of lysine 14 (K14) for further validation using AQUA approach. Our aim was to evaluate whether HIV infection and Meth treatment induces differences in Ac of lysine 14 (K14) of the human histone H3 in hMDM treated with Meth after HIV infection (CIM), before and after HIV infection (MIM), and control that was only HIV infected (CIC) (Fig. 2). This experimental design enabled us to monitor the effect of Meth on already HIV-infected hMDMs. Choosing the right approach to measure the selected PTMs together with the method development and validation for its reliable detection and quantification is itself an analytical challenge. The levels of hPTMs of HIV-infected hMDM in response to drugs of abuse, such as Meth, have had limited investigation. However, the evidence from other studies on histone acetylation show that these modifications occur in low abundance, and their quantification requires very sensitive detection methods. Thus, according to suggestions from the literature [17], we chose the AQUA proteomic approach to quantify the amounts of the K14 acetylated and unmodified fractions of human histone H3 in CIC, CIM, and MIM donors’ samples.

### LC-M/MS method development with SIL peptide standards for targeted absolute quantification of H3 K14Ac in hMDM

AQUA is a targeted MRM-based approach where the absolute quantities of the peptides originating from the protein of interest are calculated by comparing the peak area ratios of their simultaneously analyzed in the same LC–MS/MS run SIL standards of known concentrations [34]. The SIL peptides have the same amino acid sequence as the analyzed unlabeled ones, thus the same performance in the LC–MS/MS analysis. However, the stable isotopes (^13^C, ^15^N) are incorporated in one or more amino acids that result in the mass shift of the SIL peptide(s), therefore, distinguishing them from unlabeled native peptides in mass spectrometry analysis. In our case the C-terminal arginine (R) of the SIL standards of the analyzed endogenous peptides was ^13^C-^15^N labelled and doubly charged, resulting in mass shift of 5 m/z units (10 Da) (Table 1).

For protein digestion, we used trypsin that cleaves after lysine and arginine. In the presence of lysine modification, trypsin typically does not cleave. In the AQUA methodology, protein quantities are calculated based on their proteotypic peptides quantities. Therefore, to be able to compare K14 modified and unmodified on histone H3, we had to propionylate free lysines to block them from trypsin cleavage. In this way, in the sample preparation process, we obtained the peptides of the same length and sequence that differed by K9 and K14 modification. The K9 was propionylated (Poy) in both, acetylated and non-acetylated peptide, while the K14 was acetylated (Ac) or propionylated (Poy) in modified and non-modified peptides, respectively. Therefore, the peptides quantified in this study and their SIL standards were: K9[Poy]K14[Ac], and K9[Poy]K14[Ac]-heavy for acetylated K14 and K9[Poy]K14[Poy], and K9[Poy]K14[Poy]-heavy for unmodified K14 (lysines were only blocked by propionylation, Poy). The LC–MS/MS method for their quantification was developed using standard solutions of the custom-synthetized native light and reference heavy peptides.

### Validation of the AQUA method

To validate the LC–MS/MS method developed for absolute quantification of the K9[Poy]K14[Ac] and K9[Poy]K14[Poy] peptides in CIC, CIM, and MIM hMDM samples with the use of the SIL standard counterparts of these endogenous peptides, we chose fit-for-purpose approach [32] and based on the recommendations included in [27] of the CPTAC of National Cancer Institute at the National Institutes of Health. The guidelines were prepared with a purpose of validation of the assays for targeted peptide measurements using mass spectrometry in research and clinical setups, which focus on the fields of biology and medicine [32]. Our target analytes—endogenous histone H3 peptides K9[Poy]K14[Poy]APR and K9[Poy]K14[1Ac]APR were likely to occur in significant amounts in the hMDM histone sample matrix. Therefore, we prepared reversed calibration curves, where the heavy labeled SIL standard peptides K9[Poy]K14[Poy]APR-heavy and K9[Poy]K14[1Ac]APR-heavy were added in different concentrations to the sample matrix, while the unlabeled K9[Poy]K14[Poy]APR-light and K9[Poy]K14[1Ac]APR-light peptides were added as internal standards in constant amount (Additional file 2: Figures S1 and S2). The linear ranges for both analytes, the H3 peptide heavy peptides K9[Poy]K14[Poy]APR-heavy and K9[Poy]K14[1Ac]APR-heavy, were from 0.5 to 2500 fmol/µL, with their respective R^2^ values of 0.9998 and 0.9997 (Additional file 1: Table S2). The calculated accuracy for K9[Poy]K14[Poy]APR-heavy peptide was between 93.2% to 111% and precision was ≤ 5.48%. With regard to K9[Poy]K14[1Ac]APR-heavy peptide, the accuracy was between 88.9% and 112.5%, while precision was within 4.85% (Additional file 1: Table S2). This indicates good linearity in the sufficient range of the proposed AQUA LC–MS/MS method (Table 2).

**Table 2:** Summary of the validation results of the developed LC–MS/MS method in MRM mode

Validation parameter	K9[Poy]K14[Poy]-heavy peptide	K9[Poy]K14[Ac]-heavy peptide
LLOD	0.106 fmol/µL	0.204 fmol/µL
LLOQ	0.5 fmol/µL	0.5 fmol/µL
ULOQ	2500 fmol/µL	2500 fmol/µL
Accuracy	93.2–111%	88.9–112.5%
Precision	0.44–5.48% (%CV)	0.83–4.85% (%CV)
Linearity	R^2^ = 0.9998	R^2^ = 0.9997
Linear range	0.5–2500 fmol/µL	0.5–2500 fmol/µL
Sensitivity	0.5 fmol/µL	0.5 fmol/µL
Specificity	Unique MRM transitions for K9[Poy]K14[Poy]-heavy peptide were measured	Unique MRM transitions for K9[Poy]K14[Poy]-heavy peptide were measured. MRM transitions that overlap with the isobaric K9[Ac]K14[Poy]-heavy peptide were excluded
Selectivity	No interfering signals in the region of the analyte elution	No interfering signals in the region of the analyte elution

The sensitivity of the assay was determined by LLOQ. The LLOQ for both studied peptides was 0.5 fmol/µL, as this was the lowest non-zero standard concentration used for creation of the reversed calibration curve, with variability of the assay (%CV) equal to 5.48% for peptide K9[Poy]K14[Poy]APR-heavy and 4.22% for peptide K9[Poy]K14[1Ac]APR-heavy (Additional file 1: Table S2). The LLOD, determined as 3 × SD of the blank sample, for K9[Poy]K14[Poy]APR-heavy peptide was 0.106 fmol/µL, while for K9[Poy]K14[1Ac]APR-heavy peptide was 0.204 fmol/µL (Additional file 1: Table S2). The highest standard concentration on the linear part of the reversed calibration curves for both peptides was 2500 fmol/µL; and hence, it was determined as the ULOQ (Table 2).

The method can be considered selective as the visual inspection of the MRM transitions of the matrix blank samples showed no interfering peaks in the region of the analyte’s elution (Additional file 2: Figures S3 and S4).

During the method development and to ensure specificity of the assay, we checked for the MRM transitions of K9[Poy]K14[Ac] peptide that overlap with isobaric K9[Ac]K14[Poy] peptide that might also be present in the donors’ samples and excluded them from the study. The specificity of the AQUA LC–MS/MS assay was evaluated by calculating %CV of the MRM transitions ratios. The MRM transitions of the developed assay deviated from the mean between 1.42% to 21.51%, both in standard dilutions and donors’ samples above the LOQ (Additional file 1: Table S5). We also verified that all the MRM transitions for particular analytes are overlapping, and their retention times did not shift in the standard dilutions and donors’ samples. These prove that the method ensures specific detection of the peptides of interest (Table 2).

### LC–MS/MS analysis in the MRM mode of the donors CIC, CIM and MIM samples

The developed and validated AQUA LC–MS/MS method was applied for measurements of two endogenous histone H3 peptides K9[Poy]K14[Poy] and K9[Poy]K14[Ac] and their corresponding SIL counterparts K9[Poy]K14[Poy]-heavy and K9[Poy]K14[Ac]-heavy, respectively, in the six donors’ CIC, CIM, and MIM samples. The obtained areas under the peaks were used for calculating light to heavy peak area ratios (Additional file 1: Table S4). The light to heavy peak area ratios together with information of concentration of the added heavy SIL peptide standards enabled us to calculate the absolute abundance of the native peptides in the donors’ samples in each of the investigated conditions (Additional file 1:Table S4). The absolute abundances varied between the donors and conditions. At the same time, the %CV of the calculated absolute abundance for triplicate measurements of the samples from each condition (CIC, CIM, or MIM) for each separate donor were from 0.05 to 6.4% and 0.21 to 4.55% for H3 peptides K9[Poy]K14[Poy] and K9[Poy]K14[Ac], respectively. In the case of the QC sample, the %CV was 1.26% for H3 peptide K9[Poy]K14[Poy] and 0.70% for H3 peptide K9[Poy]K14[Ac] (Table 2). This indicates low variability between the replicate measurements of the same samples and shows that the developed AQUA LC–MS/MS assay provides reliable results (Additional file 1: Table S4).

### Stoichiometry of histone H3 lysine K14 acetylation (Ac) in CIC, CIM and MIM hMDM macrophages

**Fig. 3:**
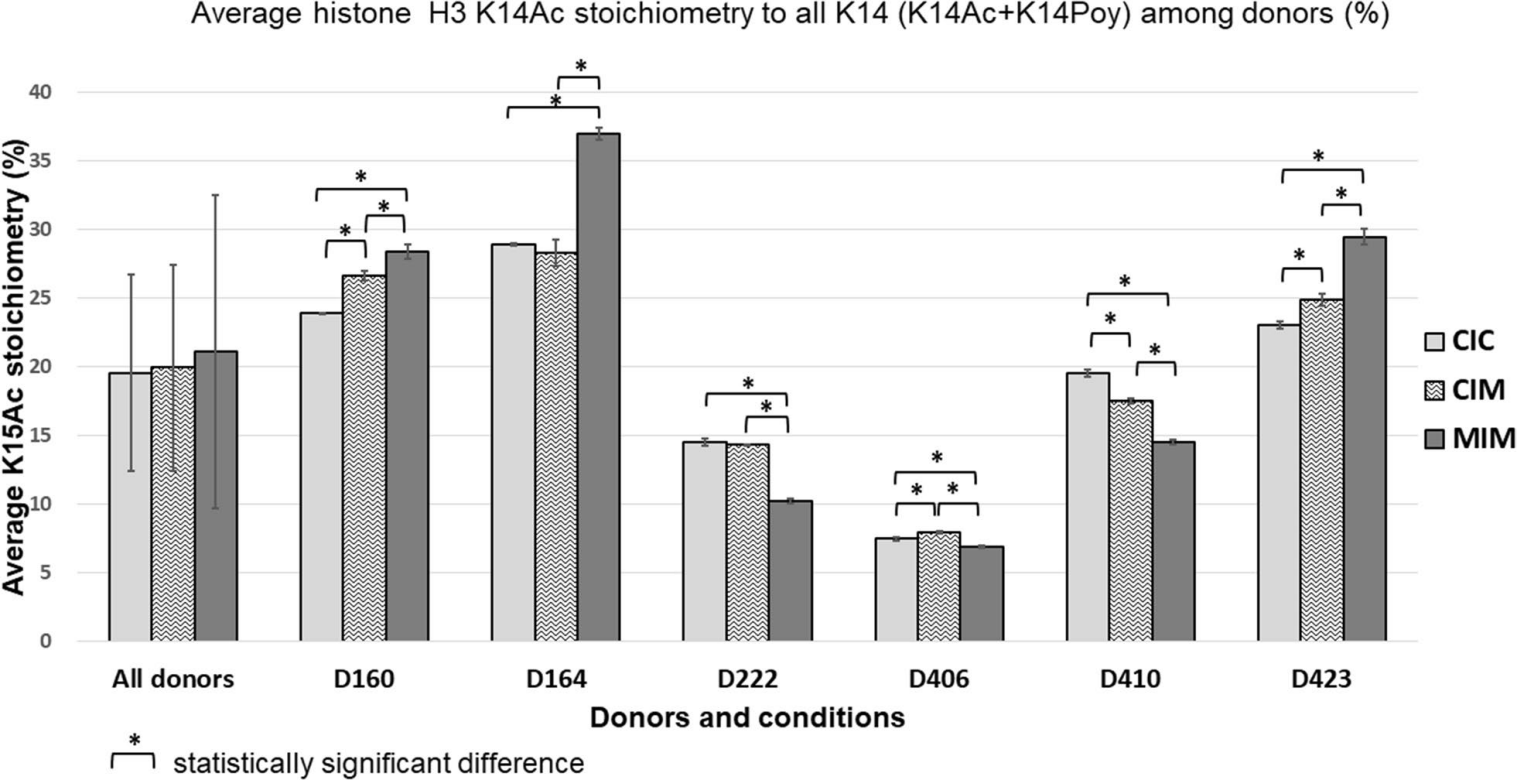
Histone H3 K14Ac stoichiometry in the donors’ samples

In this study, we were interested in measurements of how histone H3 K14Ac changes occurring due to the exposure of primary macrophages to Meth. The absolute quantification of the histone H3 K9[Poy]K14[Poy] and K9[Poy]K14[Ac] peptides in the donors’ samples enabled us to determine the absolute abundance of these peptides in each separate donor sample and in each of the three conditions tested (CIC, CIM, and MIM). Based on the absolute abundances of the native modified K9[Poy]K14[Ac] and unmodified K9[Poy]K14[Poy] peptides, we calculated stoichiometry of the K14Ac in the donors’ samples as a percentage of total amount—a sum of K14 modified (acetylated, Ac) and unmodified (propionylated, Poy) fractions—of the investigated H3 peptide [17]. Although PTMs stoichiometry can be was determined using label free, relative MS approaches (eg [35].), we chose to calculate it based on the absolute amounts of modified and unmodified peptides determined in LC–MS/MS analysis in MRM mode using SIL standards. We chose this approach, because the H3K14Ac peptide investigated in our study was showed to have 1.8 fold decreased MS detection efficiency comparing to unmodified peptide. This can negatively impact the stoichiometry calculations of this hPTM [36]. SIL standards will correct for differences in LC–MS performance of the peptides, thus enabling us to obtain possibly the most reliable results [32, 37]. We observed significant variability of responses between the donors and investigated conditions (CIC, CIM, MIM), both in the K9[Poy]K14[Ac] and K9[Poy]K14[Poy] peptides absolute abundances, and, hence in the K14Ac stoichiometry as shown in Fig. 3 and Additional file 1: Table S6. We could observe two patterns. One, observed in donors D160, D164 and D423, was a statistically significant increase in the K14Ac stoichiometry in MIM compared to CIC and MIM compared to CIM. For two donors this pattern was also characterized by the statistically significant increase in K14Ac stoichiometry in CIM in comparison to CIC. However, in one donor (D164) the K14Ac slightly decreased in CIM compared to CIC, but this change was not statistically significant. In the case of the three other donors (D222, D406, D410), an opposite pattern was observed, where the K14Ac stoichiometry was decreasing in MIM compared to CIC and CIM. The change of K14Ac stoichiometry in CIC to CIM conditions in these donors was inconsistent. We observed a decrease of K14Ac in CIM to CIC, although only in one case (D410) this relation was statistically significant. On the other hand, in D406 the stoichiometry of K14Ac showed a statistically significant increase in CIM compared to CIC. When all together we compared the CIC, CIM, and MIM conditions for all donors included in this, we observed high CV in the % of the K14Ac stoichiometry for each of these conditions (Fig. 3). Because of this, the comparisons of the studied conditions between each other, namely CIC vs. CIM, CIC vs. MIM, and CIM vs. MIM, for all the investigated donors together did not show statistically significant changes. However, we could observe an increasing trend in K14Ac stoichiometry from CIC to CIM and from CIM to MIM that was mainly contributed by overall higher K14Ac stoichiometry in donors D160, D164, and D423.

## Discussion

In this work, we focused on change in acetylation (Ac) of lysine 14 (K14) in Histone H3.At first we focused on development and validation AQUA LC–MS/MS method and tested how it can be used to investigate changes in hMDM. The obtained absolute quantities of modified and acetylated H3 peptide 3–17 enabled us to calculate stoichiometry of this PTM in HIV-infected and/or Meth treated at different experimental points hMDM. Ac influences transcription regulation leading to many intracellular effects. Lysine residues are positively charged on the side chains while acetylation cause neutralization of the charge. This leads to the change in negatively charged DNA and positively charged histones. Thus, we may expect that modification of H3 K14Ac is participating in cell cycle regulation, cell proliferation, and apoptosis cellular differentiation, DNA replication and repair, nuclear import, and neuronal repression [38].

Current mass spectrometry-based discovery proteomic approaches have enabled comprehensive mapping of various posttranslational modifications in thousands different sites [17, 23]. As in the research of other groups on different cell types, such as stem cells (e.g. [23, 39]), the results of our previous studies also showed that histone H3 is extensively post-translationally modified [22] (Fig. 1) where we mapped the PTMs landscape of hMDM histones in the resting state [18]. It needs to be noted that some modifications might be at a very low level and can go undetected due to lack of sufficient sensitivity. A continuous increase of sensitivity of mass spectrometry and sample preparations allow us to discover those PTMs changing at very low levels as well as when changes are very small. In this paper, we were interested in quantifying changes in Ac of H3K14Ac due to exposure to Meth. As the literature shows, changes of this PTM were present in various cellular systems under changing conditions including in rodent cells under exposure to Meth [40]. We developed an AQUA-based quantitative LC–MS/MS assay in our study for quantification of the absolute amounts of histone H3 K9[Poy]K14[Poy] and K9[Poy]K14[Ac] peptides in a hMDM matrix, which was applied to calculate the stoichiometry of the H3 K14Ac. The AQUA LC–MS/MS assay enables reliable absolute quantification over the linear concentration range of 0.5 to 2500 fmol/µL of the histone H3 K9[Poy]K14[Poy] and K9[Poy]K14[Ac] peptides (R^2^ 0.9998 and 0.9997, respectively) (Table 2). We applied this assay successfully to measure absolute abundances of the histone H3 K9[Poy]K14[Poy] and K9[Poy]K14[Ac] peptides in hMDM samples obtained from six donors that were HIV-infected only (control-infected-control, CIC), Meth exposed after HIV infection (control-infected-Meth, CIM), and exposed to Meth pre- and post- HIV infection (Meth-infected-Meth, MIM), and to calculate the stoichiometry of the H3 K14Ac in the respective conditions for those donors (Fig. 2). This AQUA LC–MS/MS assay can be also applied for quantification of histone H3 K9[Poy]K14[Poy] and K9[Poy]K14[Ac] peptides, and thus, the calculation of stoichiometry of H3 K14Ac in hMDMs in other studies that focus on evaluation of the effect of different treatment or physiological state on H3 K14Ac.

Our previous studies, along with results shown in Fig. 3, showed that human primary cells present a variety of responses; thus, averaging results from several donors has big variability and does not pass scrutiny of statistical significance. On the other hand, when each donor of hMDM is analyzed individually, changes induced by Meth become significant. Since all experimental conditions were the same for cell from each donor, we postulate, as we did before, that the response would be reflected by the state of the cells at the time of collection rather than further experimental manipulation. One way to answer this question would be to perform measurements in time course type of experiments which should be done in subsequent studies.

## Conclusions

Our main conclusion from this study is that AQUA can be successfully used for measurements of hPTMs. As we targeted only single modification of one H3 residue, we only can speculate that more than one residue and/or modification can be measured in one analytical analysis. Our second conclusion shows the differences in responses between donors. We reported this previously with Rab proteins [12]. We would like to point that all measurements in primary cells are related to a single time point when cells are collected. As we postulated before, time course studies are necessary when regulatory proteins are measured. Their modification(s) might be short lasting leading to observations like we present here.

### Supplementary Information

**Additional file 1: Table S1.** The MRM transitions (Q1/Q3) monitored for histone H3 K9[Poy]K14[Poy], K9[Poy]K14[Poy]-heavy, K9[Poy]K14[Ac] and K9[Poy]K14[Ac]-heavy peptides investigated in the present study, with manually optimized declustering potential (DP), collision energy (CE) and cell exit potential (CXP). **Table S2**. LC-MS/MS method validation – peptide results. **Table S3.** LC-MS/MS method validation – transitions results. **Table S4.** The LC-MS/MS results for all investigated donors' CIC, CIM and MIM samples with absolute abundances of the endogenous K9[Poy]K14[Ac] and K9[Poy]K14[Poy]. **Table S5.** MRM transition ratios for standard dilutions and donors’ samples. **Table S6.** Stoichiometry of H3K14[Ac] in donors’ CIC, CIM and MIM samples.

**Additional file 2: Figure S1.** Reversed calibration curve for K[Poy]STGGK[Ac]APR-heavy peptide. **Figure S2.** Reversed calibration curve for K[Poy]STGGK[Poy]APR-heavy peptide. **Figure S3.** MRM chromatograms for K[Poy]STGGK[Ac]APR-heavy peptide from matrix blank spiked with 50fmol/µL K[Poy]STGGK[Ac] peptide. **Figure S4.** MRM chromatograms for K[Poy]STGGK[Poy]APR-heavy peptide from matrix blank spiked with 50fmol/µL K[Poy]STGGK[Poy] peptide.

## Data Availability

The raw MRM data from the presented study was deposited in PASSEL repository (PeptideAtlas SRM Experiment Library) and is available under the accession number PASS03789 (https://db.systemsbiology.net/sbeams/cgi/PeptideAtlas/PASS_View?identifier=PASS03789).

## Abbreviations

Abs^native_mod^: Absolute abundance of native modified peptide
Abs^native_unmod^: Absolute abundance of native unmodified peptide
Ac: Acetylation
ACN: Acetonitrile
CE: Collision energy
AQUA: Absolute quantification
CAD: Collision gas
CIC: Control-infected-control
CIM: Control-infected-Meth exposed
CPTAC: Clinical Proteomic Tumor Analysis Consortium
CV: % Coefficient of variation
CXP: Cell exit potential
DP: De-clustering potential
EP: Entrance potential
EPI: Enhanced product ion
H3: Histone H3
H3K14Ac: Histone H3 lysine 14 acetylation
HIV: Human immunodeficiency virus
hMDM: Human monocyte-derived macrophages
hPTMs: Post-translational modifications of histones
K14: Lysine 14
LC–MS/MS: Liquid chromatography-tandem mass spectrometry
LLOD: Lower limit of detection
LLOQ: Lower limit of quantification
Meth: Methamphetamine
MIM: Meth exposed-infected-Meth exposed
MP: Mononuclear phagocytes
MRM: Multiple reaction monitoring
MS: Mass spectrometry
NIB: Nuclear isolation buffer
Poy: Propionylation
PTM: Post-translational modification
QC: Quality control
RP-HPLC: Reversed-phase high performance liquid chromatography
SD: Standard deviation
SIL: Stable isotope labeled
SILAC: Stable isotope labelled amino acids in cell culture
SRM: Selected reaction monitoring
TMT: Tandem mass tags
UHPLC: Ultra-high-performance liquid chromatography
ULOQ: Upper limit of quantification

## References

[1] Winter S, Fischle W (2010). Epigenetic markers and their cross-talk. Essays Biochem.

[2] Stamos JL, Weis WI (2013). The -catenin destruction complex. Cold Spring Harb Perspect Biol.

[3] Rhodes C, Lin C-H (2023). Role of the histone methyltransferases Ezh2 and Suv4-20h1/Suv4-20h2 in neurogenesis. Neural Regen Res.

[4] Wang L, Sanchez J, Hess D, Matthias P (2023). Immunoprecipitation of HDAC6 and interacting proteins.

[5] Ghavami S, Zamani M, Ahmadi M, Erfani M, Dastghaib S, Darbandi M (2022). Epigenetic regulation of autophagy in gastrointestinal cancers. Biochim Biophys Acta (BBA) Mol Basis Dis.

[6] Khadela A, Chavda VP, Postwala H, Shah Y, Mistry P, Apostolopoulos V (2022). Epigenetics in tuberculosis: immunomodulation of host immune response. Vaccines.

[7] Church MC, Workman JL, Suganuma T (2021). Macrophages, metabolites, and nucleosomes: chromatin at the intersection between aging and inflammation. Int J Mol Sci.

[8] Kantor B, Ma H, Webster-Cyriaque J, Monahan PE, Kafri T (2009). Epigenetic activation of unintegrated HIV-1 genomes by gut-associated short chain fatty acids and its implications for HIV infection. Proc Natl Acad Sci USA.

[9] Passaro RC, Pandhare J, Qian HZ, Dash C (2015). The complex interaction between methamphetamine abuse and HIV-1 pathogenesis. J Neuroimmune Pharmacol.

[10] Basova LV, Vien W, Bortell N, Najera JA, Marcondes MCG (2022). Methamphetamine signals transcription of IL1β and TNFα in a reactive oxygen species-dependent manner and interacts with HIV-1 Tat to decrease antioxidant defense mechanisms. Front Cell Neurosci.

[11] Chilunda V, Weiselberg J, Martinez-Meza S, Mhamilawa LE, Cheney L, Berman JW (2022). Methamphetamine induces transcriptional changes in cultured HIV-infected mature monocytes that may contribute to HIV neuropathogenesis. Front Immunol.

[12] Macur K, Zieschang S, Lei S, Morsey B, Jaquet S, Belshan M (2021). SWATH-MS and MRM: quantification of Ras-related proteins in HIV-1 infected and methamphetamine-exposed human monocyte-derived macrophages (hMDM). Proteomics.

[13] Niu M, Morsey B, Lamberty BG, Emanuel K, Yu F, León-Rivera R (2020). Methamphetamine increases the proportion of siv-infected microglia/macrophages, alters metabolic pathways, and elevates cell death pathways: a single-cell analysis. Viruses.

[14] Witze ES, Old WM, Resing KA, Ahn NG (2007). Mapping protein post-translational modifications with mass spectrometry. Nat Methods.

[15] Kirkpatrick DS, Gerber SA, Gygi SP (2005). The absolute quantification strategy: a general procedure for the quantification of proteins and post-translational modifications. Methods.

[16] Kettenbach AN, Rush J, Gerber SA (2011). Absolute quantification of protein and post-translational modification abundance with stable isotope-labeled synthetic peptides. Nat Protoc.

[17] Prus G, Hoegl A, Weinert BT, Choudhary C (2019). Analysis and interpretation of protein post-translational modification site stoichiometry. Trends Biochem Sci.

[18] Olszowy P, Donnelly MR, Lee C, Ciborowski P (2015). Profiling post-translational modifications of histones in human monocyte-derived macrophages. Proteome Sci.

[19] Wang Y, Kallgren SP, Reddy BD, Kuntz K, López-Maury L, Thompson J (2012). Histone H3 lysine 14 acetylation is required for activation of a DNA damage checkpoint in fission yeast. J Biol Chem.

[20] Heller EA, Hamilton PJ, Burek DD, Lombroso SI, Peña CJ, Neve RL (2016). Targeted epigenetic remodeling of the cdk5 gene in nucleus accumbens regulates cocaine- and stress-evoked behavior. J Neurosci.

[21] Grabowska K, Macur K, Zieschang S, Zaman L, Haverland N, Schissel A (2022). HIV-1 and methamphetamine alter galectins -1, -3, and -9 in human monocyte-derived macrophages. J Neurovirol.

[22] Gendelman HE, Orenstein JM, Martin MA, Ferrua C, Mitra R, Phipps T (1988). Efficient isolation and propagation of human immunodeficiency virus on recombinant colony-stimulating factor I-treated monocytes. J Exp Med.

[23] Sidoli S, Lin S, Xiong L, Bhanu NV, Karch KR, Johansen E (2015). Sequential window acquisition of all theoretical mass spectra (SWATH) analysis for characterization and quantification of histone post-translational modifications. Mol Cell Proteomics.

[24] Hoofnagle AN, Whiteaker JR, Carr SA, Kuhn E, Liu T, Massoni SA (2016). Recommendations for the generation, quantification, storage, and handling of peptides used for mass spectrometry-based assays. Clin Chem.

[25] Whiteaker JR, Halusa GN, Hoofnagle AN, Sharma V, MacLean B, Yan P (2016). Using the cptac assay portal to identify and implement highly characterized targeted proteomics assays. Methods Mol Biol.

[26] Song E, Gao Y, Wu C, Shi T, Nie S, Fillmore TL (2017). Targeted proteomic assays for quantitation of proteins identified by proteogenomic analysis of ovarian cancer. Sci Data.

[27] Assay Development Guidelines CPTAC Assay Development Working Group Overview of assay characterization for the CPTAC assay portal CPTAC Assay Development Working Group. https://proteomics.cancer.gov/sites/default/files/assay-characterization-guidance-document.pdf. Accessed 10 Feb 2021.

[28] Farrah T, Deutsch EW, Kreisberg R, Sun Z, Campbell DS, Mendoza L (2012). PASSEL: the PeptideAtlas SRMexperiment library. Proteomics.

[29] Henderson CM, Shulman NJ, MacLean B, MacCoss MJ, Hoofnagle AN (2018). Skyline performs as well as vendor software in the quantitative analysis of serum 25-hydroxy vitamin D and vitamin D binding globulin. Clin Chem.

[30] MacLean B, Tomazela DM, Shulman N, Chambers M, Finney GL, Frewen B (2010). Skyline: an open source document editor for creating and analyzing targeted proteomics experiments. Bioinformatics.

[31] UniProt. https://www.uniprot.org/uniprotkb. Accessed 4 Nov 2021.

[32] Carr SA, Abbatiello SE, Ackermann BL, Borchers C, Domon B, Deutsch EW (2014). Targeted peptide measurements in biology and medicine: best practices for mass spectrometry-based assay development using a fit-for-purpose approach. Mol Cell Proteomics.

[33] Baker PR, Clauser KR. ProteinProspector. http://prospector.ucsf.edu. Accessed 21 Nov 2021.

[34] Gerber SA, Rush J, Stemman O, Kirschner MW, Gygi SP (2003). Absolute quantification of proteins and phosphoproteins from cell lysates by tandem MS. Proc Natl Acad Sci USA.

[35] Verhelst S, Van Puyvelde B, Willems S, Daled S, Cornelis S, Corveleyn L (2022). A large scale mass spectrometry-based histone screening for assessing epigenetic developmental toxicity. Sci Rep.

[36] Lin S, Wein S, Gonzales-Cope M, Otte GL, Yuan ZF, Afjehi-Sadat L (2014). Stable-isotope-labeled histone peptide library for histone post-translational modification and variant quantification by mass spectrometry. Mol Cell Proteomics.

[37] Bronsema KJ, Bischoff R, Van de Merbel NC (2012). Internal standards in the quantitative determination of protein biopharmaceuticals using liquid chromatography coupled to mass spectrometry. J Chromatogr B Analyt Technol Biomed Life Sci.

[38] Roth SY, Denu JM, Allis CD (2001). Histone acetyltransferases. Annu Rev Biochem.

[39] Zijlmans DW, Talon I, Verhelst S, Bendall A, Van Nerum K, Javali A (2022). Integrated multi-omics reveal polycomb repressive complex 2 restricts human trophoblast induction. Nat Cell Biol.

[40] Xie F, Li BX, Kassenbrock A, Xue C, Wang X, Qian DZ (2015). Identification of a potent inhibitor of CREB-mediated gene transcription with efficacious in vivo anticancer activity. J Med Chem.

