# Supplementary material for: Change of histone H3 lysine 14 acetylation stoichiometry in human monocyte derived macrophages as determined by MS-based absolute targeted quantitative proteomic approach: HIV infection and methamphetamine exposure
Source: Clin Proteomics. 2023 Oct 25;20:48. doi: 10.1186/s12014-023-09438-5 (PMC10599040; doi:10.1186/s12014-023-09438-5)
Supplement: Supplementary file 2 — Additional file 2: Figure S1. Reversed calibration curve for K[Poy]STGGK[Ac]APR-heavy peptide. Figure S2. Reversed calibration curve for K[Poy]STGGK[Poy]APR-heavy peptide. Figure S3. MRM chromatograms for K[Poy]STGGK[Ac]APR-heavy peptide from matrix blank spiked with 50fmol/µL K[Poy]STGGK[Ac] peptide. Figure S4. MRM chromatograms for K[Poy]STGGK[Poy]APR-heavy peptide from matrix blank spiked with 50fmol/µL K[Poy]STGGK[Poy] peptide. [file 12014_2023_9438_MOESM2_ESM.docx]

**Change of histone H3 lysine 14 acetylation stoichiometry in human monocyte derived macrophages as determined by MS-based absolute targeted quantitative proteomic approach: HIV infection and methamphetamine exposure.**

Katarzyna Macur^1,2*,^ Andrew Schissel^2^, Fang Yu^4^, Shulei Lei^2^, Brenda Morsey^2,3†^, Howard S. Fox^2,3^, Pawel Ciborowski^2*^

1. Core Facility Laboratories, Intercollegiate Faculty of Biotechnology UG & MUG, University of Gdańsk, Gdańsk, Poland

2. Department of Pharmacology and Experimental Neuroscience, University of Nebraska Medical Center, Omaha, NE, USA

3. Department of Neurological Sciences, University of Nebraska Medical Center, Omaha, NE, USA

4. Department of Biostatistics, University of Nebraska Medical Center, Omaha, NE, USA

† See Authors’ Contribution Section

*Corresponding author


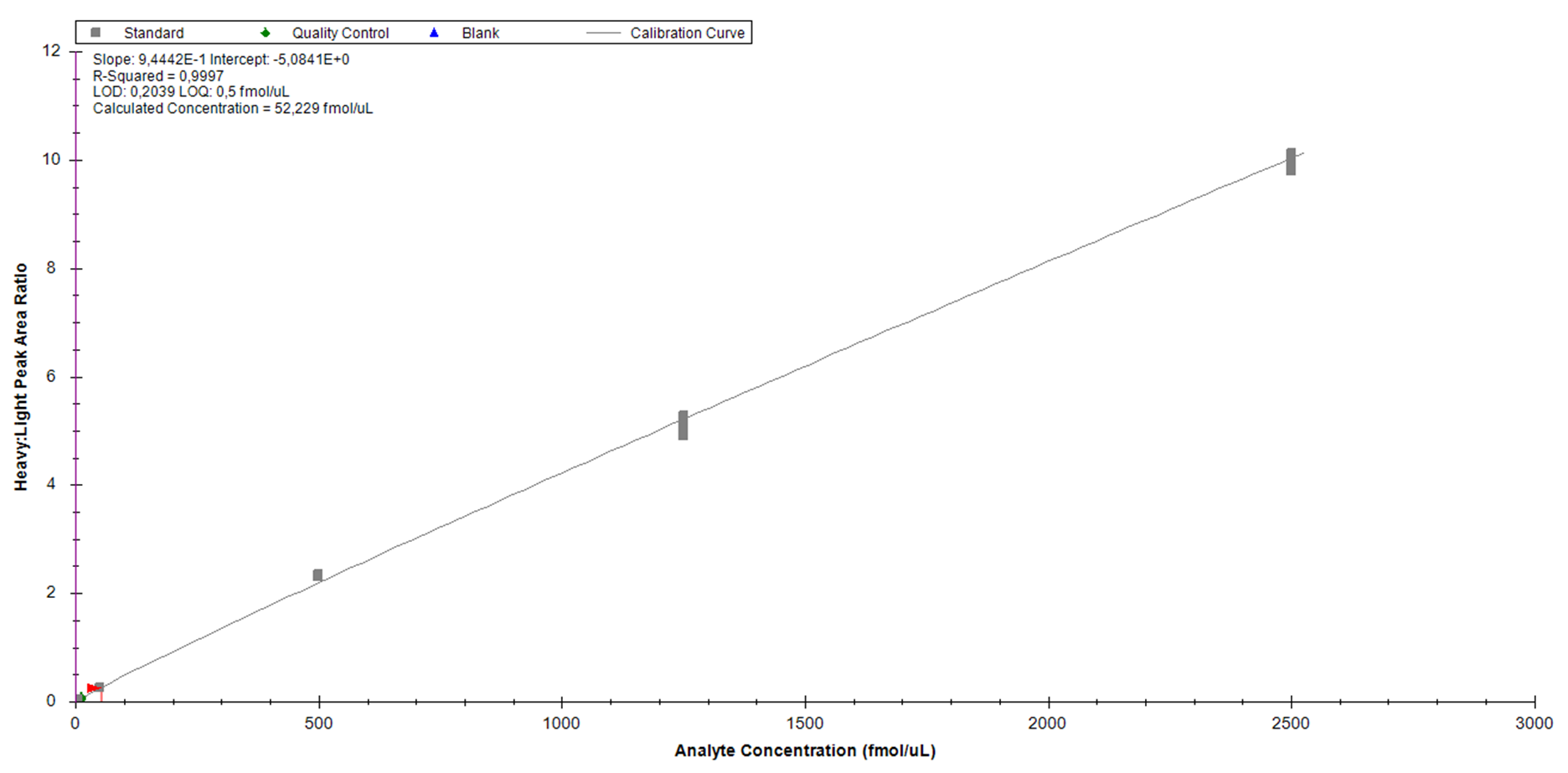


**Figure 1S.** Reversed calibration curve for K[Poy]STGGK[Ac]APR-heavy peptide.


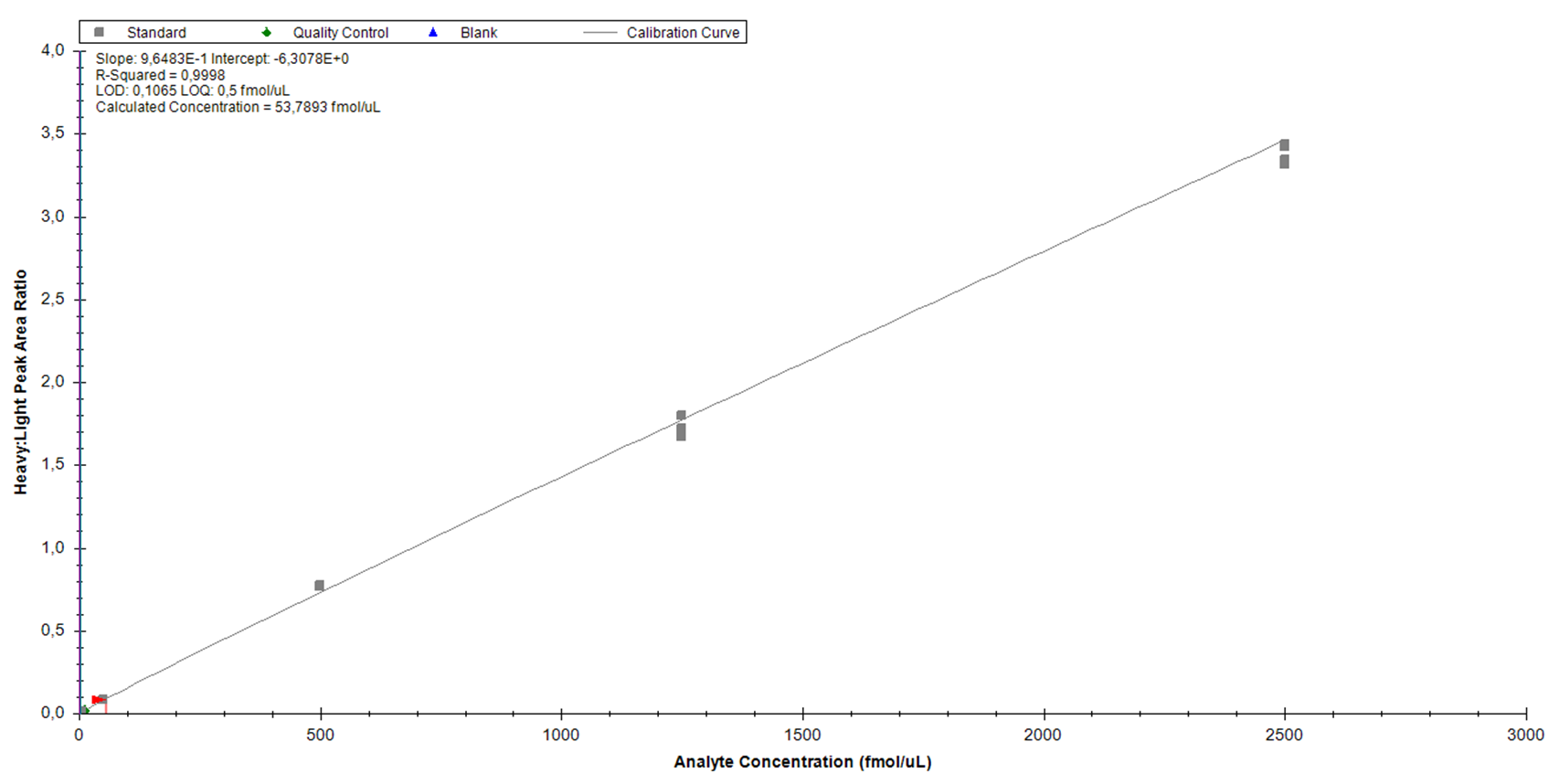


**Figure 2S.** Reversed calibration curve for K[Poy]STGGK[Poy]APR-heavy peptide.


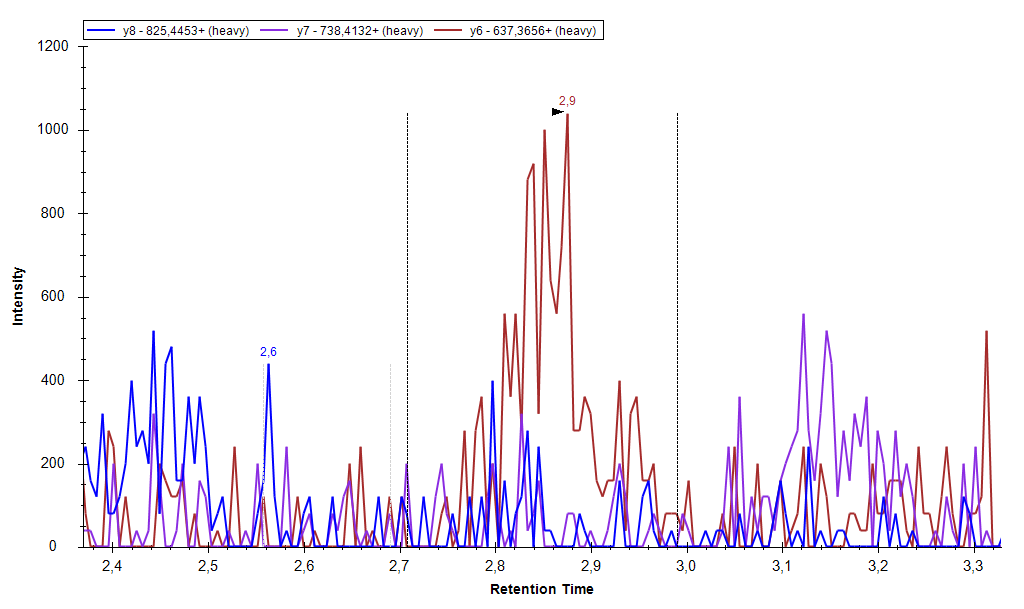


**Figure 3S.** MRM chromatograms for K[Poy]STGGK[Ac]APR-heavy peptide from matrix blank spiked with 50fmol/µL K[Poy]STGGK[Ac] peptide.


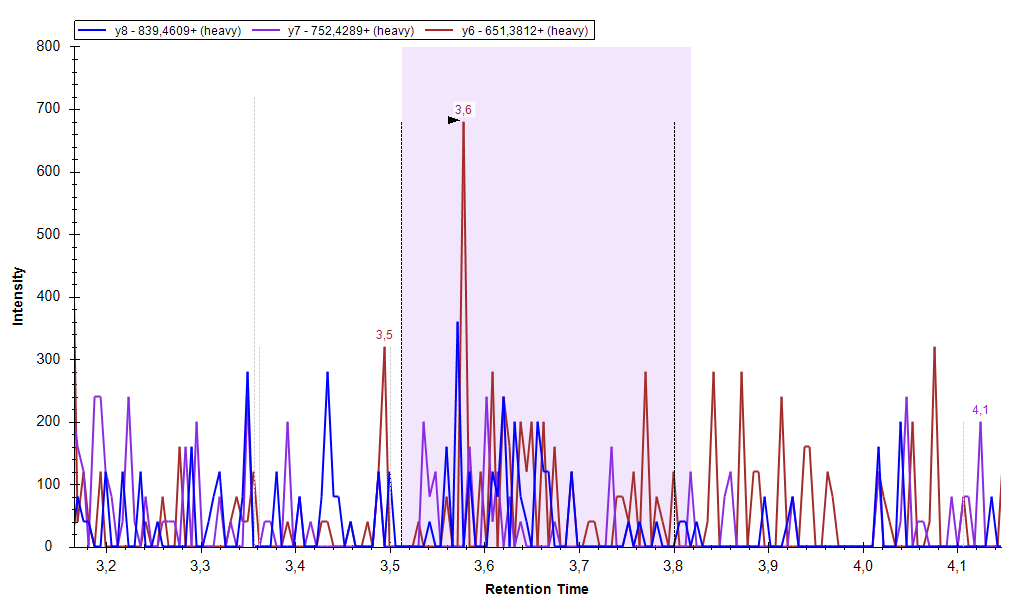


**Figure 4S.** MRM chromatograms for K[Poy]STGGK[Poy]APR-heavy peptide from matrix blank spiked with 50fmol/µL K[Poy]STGGK[Poy] peptide
